# Heatwave-protective knowledge and behaviour among urban populations: a multi-country study in Tunisia, Georgia and Israel

**DOI:** 10.1186/s12889-021-10865-y

**Published:** 2021-05-01

**Authors:** Joris Adriaan Frank van Loenhout, Kirsten Vanderplanken, Tamari Kashibadze, Nia Giuashvili, Amiran Gamkrelidze, Maya Siman-Tov, Bruria Adini, Debarati Guha-Sapir

**Affiliations:** 1 Centre for Research on the Epidemiology of Disasters, Institute of Health and Society, Université Catholique de Louvain, Clos Chapelle-aux-Champs 30, 1200 Brussels, Belgium; 2 L. Sakvarelidze National Center for Disease Control and Public Health (NCDC), Ministry of IDP from the occupied territories, Labour, Health and Social Affairs of Georgia, Tbilisi, Georgia; 3 Emergency Management and Disaster Medicine department, School of Public Health, Sackler Faculty of Medicine, Tel Aviv University, Tel Aviv, Israel

**Keywords:** Heatwave, Climate change, Risk perception, Urban population, Protective knowledge, Behaviour

## Abstract

**Background:**

There is an expected increase in heatwaves globally. As such, it is imperative to have sufficient levels of heatwave-protective knowledge and behaviour in areas regularly affected by heatwaves. Our study assessed this among urban populations in Tunisia, Georgia and Israel.

**Methods:**

We undertook a cross-sectional population survey in the three countries. The questionnaire focused on obtaining information on respondents’ knowledge level regarding 1) symptoms due to overheating, 2) risk groups for heatwaves, 3) actions to take when someone is overheated, and 4) heatwave-protective measures. Furthermore, we asked respondents about protective measures they applied during the last heatwave. We compared the results between the countries.

**Results:**

Heatwave-protective knowledge was highest in Israel, and lowest in Georgia, for all indicators except for heatwave-protective measures, for which knowledge was highest in Tunisia. Most respondents who named certain protective measures had also applied these during the last heatwave: more than 90% for all measures except for one in Tunisia and Israel, and more than 80% for all measures in Georgia.

**Conclusion:**

There is a need to further improve heatwave-protective knowledge in Tunisia, Georgia and Israel. One potential solution to achieve this is by implementing a National Heat Health Action Plan. Improving knowledge is a vital step before adaptive behaviour can take place.

**Supplementary Information:**

The online version contains supplementary material available at 10.1186/s12889-021-10865-y.

## Background

Heatwaves are climate events in which the weather is excessively hot for a prolonged period. In recent years, there has been an increase in the number of reported heatwaves globally [1]. In addition, the Intergovernmental Panel for Climate Change (IPCC) has stipulated that temperatures are set to increase even further in the coming century [2], a development that warrants urgent action. Especially in cities, the temperature increase is disproportionately high due to the urban heat island effect. Building materials, such as concrete, retain heat during the day and release it in the evening. This can lead to differences in temperatures between urban and rural settings in close proximity up to 12 °C [3].

Heatwaves have a detrimental effect on human populations. Studies have shown a relationship between heatwaves and mortality [4, 5], as well as between heatwaves and hospital admissions [6]. However, this reflects only the tip of the iceberg in terms of human impact, as the overall majority of people affected by heatwaves will not seek medical attention for their health problems. Certain groups of people are considered more at risk for health problems during heatwaves, including the elderly, infants and children, pregnant women, outdoor and manual workers, people who take certain types of medication, those who perform high-level exercise (athletes) and people who are overweight [7]. A study on heat exposure among elderly showed that almost half the participants reported health symptoms such as headache, fatigue, thirst and excessive sweating during a week with high temperatures [8]. Similarly, a study involving outdoor workers in Slovenia and Greece reported that more than 50% of participants experienced thirst, excessive sweating and exhaustion as a result of heat exposure [9].

The negative health impacts of heatwaves are predictable and largely preventable with specific public health actions. Such actions can be aimed at increasing knowledge, and consequently behaviour, of the general population on heat-protection. Specifically, knowledge on groups vulnerable to heat, recognition of heat-related health symptoms and familiarity with protective measures are key in reducing the health impact of heatwaves on individuals, as well as on vulnerable people. As a result of the 2003 European heatwave, which claimed more than 70,000 lives [1], various countries in Europe have adopted National Heat Health Action Plans [10, 11]. These plans are aimed at reducing the health impact of heatwaves on populations, in part by increasing knowledge and awareness. Several studies have assessed heatwave-protective knowledge in countries where such plans exist [12, 13]. There is limited evidence on the extent to which people adapt their behaviour during heatwaves.

Parts of the world that did not experience a turning point event like Europe, including regions in Western Asia and Northern Africa that are in proximity to Europe (European Neighbourhood) are less likely to have a National Heat Health Action Plan in place. On top of that, populations of these regions are often exposed to higher temperatures than in many European countries, as well as more frequently. Because of their familiarity with heat, it is possible that populations in these countries obtain protective knowledge through other means than through official government activities, e.g. through family members and local communities. In this study, we assessed heatwave-protective knowledge among urban populations in three countries in the European Neighbourhood, namely Tunisia, Georgia and Israel, that do not have National Heat Health Action Plans. Moreover, we compared the findings between these countries, and we investigated to what extent people who have knowledge on heatwave-protective measures report to apply these in their behaviour.

## Methods

We undertook a population survey in three countries, namely Tunisia, Georgia and Israel, using a cross-sectional study design. This study was part of the SCORCH project, in which we aim to increase heatwave preparedness in the European Neighbourhood.

### Study setting

The European Neighbourhood Policy is a mechanism in which the European Union works with countries in that region to promote peace, stability and economic prosperity [14]. It consists of an Eastern Region (Armenia, Azerbaijan, Belarus, Georgia, Moldova, Ukraine) and a Southern Region (Algeria, Egypt, Israel, Jordan, Lebanon, Libya, Morocco, Palestine, Syria, Tunisia). Since the territory is extremely vast, there is ample variation between the countries in the European Neighbourhood in terms of culture, politics, and climate. In particular, the Southern Region is characterized by extreme temperatures, due to its proximity to the Sahara and Arabian desert.

We carried out this study in three countries in the European Neighbourhood, namely Tunisia, Georgia and Israel. Each of these countries is located in a different geographic region, namely Northern Africa, the Caucasus, and the Arabian Peninsula, and as a result there are remarkable differences. We provide a short overview of climatic and socio-demographic characteristics for each country, as these are most relevant for our study.

#### Tunisia

Currently, Tunisia’s population is estimated to be approximately 11.7 million people with an average life expectancy of 76.3 years [15]. The proportion of women among the total population is 50.3%. Arabic is the official language of the country. The majority of the population (99%) are Sunni Muslims with the rest following Christianity, Judaism or other religions [15]. The country’s GDP per capita was $3287 in 2019 [16]. The unemployment rate was 15.3% in the first half of 2019, and unemployment among women (22.6%) was significantly higher when compared to the overall unemployment rate [17]. The school enrolment rate for tertiary education was 32% in 2019 [17].

The climate of Tunisia is influenced by its geographical position. The North is affected by the Mediterranean Sea and has a Mediterranean climate characterized by a summer that is hot and dry, and with a relatively mild and rainy winter. The South, which is impacted by the Sahara desert, has an arid climate with high temperatures and rainfall rarely exceeding 100 mm/year. The Central region, which is under the joint influence of the Mediterranean Sea and the Sahara, has a semi-arid climate characterized by relatively high temperatures and modest rainfall between 200 and 400 mm/year [18]. Temperatures in the summer may easily reach 40 °C, and even 45 °C in the South [19].

#### Georgia

Georgia has a population of 4.0 million people with an average life expectancy of 77.0 years [20]. The proportion of women among the total population is 52.1%. Orthodox Christianity is the primary religion of the country, with 83.4% of the population being a member of the Georgian Orthodox Church. 10.7% of the population is Muslim, 2.9% Armenian Apostolic and 1.2% has another religion, including Catholicism [20]. Georgian is the official language of the country. The GDP per capita of Georgia in 2019 was $4289 [16], and the unemployment rate 11.6% [21]. The enrolment rate for tertiary education was 57% in 2017. Around 42% of the population aged 25–29 years holds a tertiary degree, compared to 27% among 60–64 year-olds [22].

Georgia’s geography is characterized by sharply expressed vertical zoning, and two thirds of the territory of Georgia is mountainous [23]. The climatic diversity of Georgia is conditioned by its geographical location, its location in the extreme north of the subtropical zone, between the Black and Caspian Seas and by the specific complexity of the relief. While the West is defined by rather mild winters and hot summers, the eastern climate is continental, with the mountain region being the coldest [24, 25]. The majority of Georgia has a subtropical climate, with summertime minimum and maximum temperatures averaging 16 and 27 °C [25]. Heatwaves are common occurrences in Georgia, reaching temperatures well over 40 °C [26].

#### Israel

Israel has a diverse ethnic population of 8.7 million residents, with an overall life expectancy of 83 years of age [27]. The proportion of women among the total population is 49.8%. Accounting for 74% of the population, Jews encompass the largest ethnic group. Israeli-Arabs make up the second largest ethnic group and account for 21% of the population [27]. Hebrew and Arabic are the official languages of Israel, but English is widely spoken as well. Although the majority of the population is Jewish, only 49% of the entire population considers Hebrew as their first language, and 18% of the Israeli population speak Arabic as a first language [28]. The GDP per capita in Israel was $42,823 in 2019 [16]. Furthermore, the Israeli labour market was close to full employment, with an unemployment rate of 3.9% [29]. Around 46% of the population aged 25–64 has achieved tertiary education [30].

Israel’s climate ranges between temperate and tropical, depending on the geographical location. It is characterized by two distinctive seasons, a rainy winter period lasting from November to May and a dry summer period lasting from June to October [31]. These seasons vary regionally with humid summers and mild winters along the coast, dry summers and moderately cold winters in the hill regions, hot dry summers and mild winters in the Jordan valley and annual semi-desert conditions in the Negev desert. Rainfall is relatively heavy in the northern and central part of the country, with much less rain falling in the northern part of the Negev desert to almost none in the southern parts of the desert [31]. Heatwaves are a common phenomenon, with temperatures above 40 °C, and sometimes nearing 50 °C [32].

### Selection of cities

In each country, we selected three cities in which we undertook the study. All selected cities are among the biggest cities in each country, and represent different parts of the country in terms of climate zones. In Tunisia, we included the capital Tunis in the North (Mediterranean climate), and Kairouan and Gafsa in the Central region (joint influence of Mediterranean and Sahara). We did not include a city in the South, since the population size there is very small. In Georgia, we included Tbilisi and Telavi in the East, both with a continental climate, and Batumi in the West, with a subtropical climate and typically hot summers. We did not include a city in the mountain region, since the population size there is small and this region is affected less by heatwaves than the rest of the country. In Israel, we selected Tel Aviv and Haifa, which are both located in the Mediterranean Coastal Plain, and Beer Sheva, which is located in the Negev desert with semi-desert climate conditions.

### Data collection

The data were collected in September 2019, by undertaking a population survey. Due to certain differences between the countries, we adapted the data collection methodology accordingly. A large share of the Israeli population has access to internet, and uses this medium widely. This is much less the case in Tunisia and Georgia, and in particular among marginal populations such as the elderly and people from low socio-economic status. As such, we decided to undertake a street survey in the latter two countries, versus an internet survey in Israel.

#### Tunisia and Georgia

We undertook a street survey in each of the selected three cities in Tunisia and Georgia. Per city, different locations were selected based on the presence of a relatively large number of passer-by’s (e.g. markets, malls, shopping streets). However, we avoided locations where people would be in a hurry, such as train or bus stations, and locations that would have a relatively low proportion of locals, such as touristic spots. In addition, we ensured variation in the selected locations per city in terms of socio-economic backgrounds, i.e. high, low and mixed.

The survey team, consisting of one data manager and several enumerators, collected the data in each location. All of them had been previously trained in undertaking the survey in a standardised way (e.g. asking questions as literal as possible, and making sure to know which questions had to be asked in an open way). Random passer-by’s were asked to participate, namely each fifth individual, to avoid inclusion of clusters of people from the same group (e.g. husband and wife). The survey started with a brief description of the study, after which the passer-by was asked for oral informed consent to participate. Only adults (≥ 18 years) living in the city of interest were eligible to participate. The duration of each survey was around 10 min. The data manager kept track of the number of completed surveys, assessed when the target number of surveys was reached and the team could move to the next location.

In Tunisia, data were collected using tablets. In Georgia, data were collected using paper surveys.

#### Israel

In Israel, an online panel was used to participate in the survey. We collaborated with iPanel, a survey company with the largest online panel of the country (> 100,000 participants). For each panellist, baseline information was known, such as gender, age, city of residence, educational level and ethnicity. This enabled us to select a sample in each of the three study cities. Only adults (≥ 18 years) were eligible to participate. The survey targeted a specific distribution between the Jewish population (80%) and the Arabic population (20%), broadly reflecting the population of Israel and covering the two main ethnic groups.

The online survey took on average 15 min. It also included quality assurance questions, to eliminate respondents that clicked e.g. the first answer in all questions.

### Sample size calculation

For each country, we aimed to obtain a sample of study participants representative for that country in terms of age and sex, with variation in composition for educational level. We calculated the required sample size using Cochran’s sample size formula:

$$ {\mathrm{n}}_0={\mathrm{Z}}^2\ast \mathrm{p}\ast \left(1-\mathrm{p}\right)/{\mathrm{e}}^2 $$

We used a margin of error (e) of 5%, a confidence interval of 95% (Z = 1.96) and a population proportion (p) of 50%, the most conservative estimate. We found that the minimal required sample size was 385. To ensure a sufficiently large sample, in case some questionnaires could eventually not be used, we decided to include a small margin and include at least 30 respondents extra per country. In Israel, where the survey was carried out over the internet, the sample was increased to a minimum of 500. The reason for this was that it would allow for an in-country comparison between different subgroups (i.e. Jews and Arabs), which is addressed in another article, currently under consideration by another journal. A stratified sampling method was used to achieve a representative sample, based on data published by the Central Bureau of Statistics of Israel in regard to age, gender, ethnicity and geographic zones. However, it was difficult to include a sufficient number of Arabic respondents who matched the demographic and geographic distribution of the population, due to which our sample had a deviation from the general population in terms of age and gender. The sample size per city was calculated based on the relative size of the population in relation to the overall country’s population: cities in administrative regions with a larger population contributed more to the survey than cities representing an administrative region with a smaller population.

### Questionnaire development

We developed a questionnaire based on a format that we previously used in four European countries, namely Netherlands, Belgium, Spain and Portugal [12, 13]. The survey can be found in Additional file 1. Socio-demographic questions were multiple choice. A question on educational level was adapted in each country to reflect the local situation. Four questions related to the heatwave-protective knowledge of respondents concerned: 1) symptoms, 2) risk groups, 3) action in case of overheating, 4) protective measures. We first asked respondents if they could name any e.g. symptoms people may experience due to heatwaves. If yes, respondents were asked openly to name all answers that they could think of. For those respondents in Tunisia and Georgia who named certain protective measures, we asked them whether they used these measures during the last summer. Similarly, respondents in Israel were first asked to name protective measures in an open question. Next, they received a pre-determined list of measures for which they had to indicate whether they used the listed measures ‘always’, ‘usually’, ‘occasionally’ or ‘never’ during the last heatwave, or whether the measure was not relevant for them. Finally, we asked respondents which information channel they had used during the last summer to obtain information on heatwave-protective measures. This was asked as an open question in Tunisia and Georgia, and as a closed question with multiple answer options and an open text option in Israel.

We developed the original version of the questionnaire in English. The final versions of the survey were translated into Tunisian Arabic (Tunisia), Georgian (Georgia), Hebrew and Arabic (Israel). A back-translation was conducted as well, to ensure the accuracy.

### Data description

Educational level in each country was reclassified into three levels, namely low, medium and high. Low educational level consists of ‘none’, ‘primary education’, ‘basic education’, and ‘secondary education’; medium educational level consists of ‘vocational/professional education’; high educational level consists of ‘college/university’. The open answers to the questions on heatwave-protective knowledge were categorized into different groups. These groups were partly predefined and partly created based on responses. The final groups were decided through a process that ensured inter-rater reliability between two researchers. Each researcher categorised the open answers according to his/her best assessment, after which they were discussed and a final agreement was made on the groups, as well as the answers belonging to each group. For the groups related to heatwave-related symptoms, we obtained additional input from a medical doctor, who checked the classification which was proposed by the researchers, and consensus was reached on which all three agreed. For all groups per question, we determined which groups reflected ‘correct’ answers, and which ones ‘incorrect’. Incorrect in this case means that the answer is not supported by current insights or literature, e.g. diabetes as a heatwave-related symptom. All correct answers per question were summed, to come up with a total number of correct answers per respondent.

### Data analysis

We carried out all analyses using IBM SPSS Statistics 25. We used Pearson’s chi-square tests and Analysis of Variance (ANOVA) to test for differences in socio-demographic characteristics, for categorical and continuous variables, respectively. We compared the average number of correct answers given by respondents between countries, using multivariable linear regression. We included the following variables as confounders in the model, as the frequencies/outcomes differed between countries and preliminary analyses had shown they were potentially related to the outcome variables [33]: gender, age, educational level, having children under 12 years old, employment status, having fasted in the previous year, and taking medication for a chronic disease. A *p*-value of < .05 was considered to be statistically significant, based on two-sided tests.

## Results

### Descriptive statistics

The population descriptives for each study country are presented in Table 1. The sample sizes were 417, 420 and 556 for Tunisia, Georgia and Israel, respectively. One questionnaire from a Georgian respondent was not completed, which made it unfit for use in the analyses and brings the final sample size to 419. There were significant differences in all personal characteristics between the study countries. Respondents in Israel were younger and more often female than in the other two countries. This was mainly because of the researchers’ decision to include a sample of Arabic respondents, for whom it was difficult to include a sample representative for the general Israeli population in terms of gender and age, as explained in the section ‘Sample size calculation’. However, the lower age among Israel respondents is in line with the demographic differences between Israel, Tunisia and Georgia (30.4, 32.7 and 38,8 for the total populations, respectively) [15, 20, 27]. The proportions of women among the samples for Tunisia and Georgia roughly correspond to the proportion of women in the general population (50.3 vs. 49.9% and 53.7 vs. 52.1%, respectively). Respondents in Tunis were on average lower educated than in the other two countries, which is in line with the overall proportion of the population enrolled in or who attained tertiary education [17, 22, 30]. In Georgia and Israel, more than two-third of the respondents were employed or self-employed, versus half in Tunisia. Unemployment rates among respondents roughly corresponded with overall country unemployment rates in Tunisia (12.3 vs. 15.3%), Georgia (7.9 vs. 11.6%) and Israel (4.5 vs. 3.9%) [17, 21, 29]. Having children under the age of 12 years was more prevalent in Israel, which is probably linked to the lower average age. Similarly, the fraction of respondents taking medication for a chronic disease was highest in Georgia, where the average age of respondents was highest as well. Of the people who were currently employed, around 50% works mainly outdoors in Tunisia and Georgia, while this was only a quarter in Israel. The proportion who work as care-givers of others was higher in Tunisia and Israel (around 20%) compared to Georgia (10%).

**Table 1:** Descriptive overview of study participants

Personal characteristics	Tunisia *N* = 417	Georgia *N* = 419	Israel *N* = 556	Difference
	N (%)	N (%)	N (%)	*p*-value
Gender				< .001
Male	209 (50.1)	194 (46.3)	202 (36.3)	
Female	208 (49.9)	225 (53.7)	353 (63.5)	
Other	0 (0.0)	0 (0.0)	1 (0.2)	
Mean age (sd)	42.3 (16.0)	48.6 (18.0)	36.3 (13.5)	< .001
City^a^				NA
*A*	231 (55.4)	265 (63.2)	233 (41.9)	
*B*	114 (27.3)	85 (20.3)	119 (21.4)	
*C*	72 (17.3)	69 (16.5)	204 (36.7)	
Education				< .001
Low	267 (64.0)	156 (37.2)	179 (32.2)	
Medium	37 (8.9)	53 (12.6)	85 (15.3)	
High	113 (27.1)	210 (50.1)	292 (52.5)	
Children below 12	89 (21.3)	111 (26.6)	182 (32.7)	< .001
Employment				< .001
Student	47 (11.3)	27 (6.4)	71 (12.8)	
Employed / self-employed	210 (50.5)	291 (69.5)	398 (71.6)	
Unemployed	51 (12.3)	33 (7.9)	25 (4.5)	
Retired	41 (9.9)	44 (10.5)	23 (4.1)	
Housewife	67 (16.1)	22 (5.3)	0 (0.0)	
Other	0 (0.0)	2 (0.5)	39 (7.0)	
Work mainly outdoors^b^	101 (50.5)	148 (50.7)	106 (26.6)	< .001
Work taking care of others^b^	42 (21.0)	29 (9.9)	86 (21.6)	< .001
Participated in (religious) fast	337 (80.8)	154 (36.8)	146 (26.3)	< .001
Medication for chronic disease	108 (25.9)	180 (43.0)	119 (21.4)	< .001

^a^Cities A, B and C represent Tunis, Kairouan and Gafsa (Tunisia); Tbilisi, Batumi and Telavi (Georgia); Tel Aviv, Beer Sheva and Haifa (Israel), respectively;

^b^The questions on ‘working mainly outdoors’ and ‘work consists of taking care of others’ were only addressed to respondents who were employed/self-employed. The sample sizes were 200, 292 and 398 in Tunisia, Georgia and Israel, respectively

### Heatwave-protective knowledge

Table 2 displays the answers on questions related to heatwave-protective knowledge that were reported by respondents, namely 1) symptoms, 2) risk groups, 3) action in case of overheating, 4) protective measures. Correct answers (see ‘data description’ section in Methodology) are listed in black, incorrect answers in italic. The widest range of correct answers was given for the question on symptoms, and the most commonly reported symptoms were health problems related to dehydration (dehydration, excessive sweating), headache, and exhaustion. For the question on risk groups, more than half of respondents were able to name elderly, and almost half named babies and/or children. Around 8% of respondents named the general population as a risk group, which we did not consider specific enough to be correct. In terms of actions to take when someone is overheated, respondents reported limited diversity in their answers, with the most common answers being hydrating the overheated person, providing medical care (first aid / calling emergency services) and placing the affected person in a cool location. Finally, concerning heat-protective measures, more than half of respondents expressed the combined answers of staying inside and visiting cool areas, which was closely followed by increased fluid consumption (drinking more) with 49%.

**Table 2:** Most prevalent heatwave-protective answers given by respondents

Question	Answer^**a**^	N (%)
Symptoms	Dehydration-related problems	537 (38.6)
	Headache	431 (31.0)
	Exhaustion	410 (29.5)
	Thermoregulation-related problems	341 (24.5)
	Dizziness / fainting	230 (16.5)
	Cardiovascular problems	203 (14.6)
	Skin problems	202 (14.5)
	Respiratory problems	169 (12.1)
	Behavioural and cognitive problems	147 (10.6)
	Gastrointestinal problems	50 (3.6)
	Neuromuscular problems	21 (1.5)
	Other general problems	17 (1.2)
	Eye problems	13 (0.9)
	*Death*^b^	*36 (2.6)*
	*Nosebleed*	*20 (1.4)*
	*Allergy*	*8 (0.6)*
	*Cancer*^c^	*8 (0.6)*
	*Diabetes*	*6 (0.4)*
	*Seizures*	*2 (0.1)*
Risk groups	Elderly	831 (59.7)
	Babies / children	671 (48.2)
	Physically ill	490 (35.2)
	Pregnant women	170 (12.2)
	People who perform physical effort / work mainly outdoors	152 (10.9)
	People who use medication for chronic disease	71 (5.1)
	Obese	39 (2.8)
	Handicapped or limited mobility	33 (2.4)
	Socially isolated	27 (1.9)
	Mentally ill	13 (0.9)
	People with lower socio-economic status	10 (0.7)
	Substance abusers	1 (0.1)
	*General public*	*112 (8.0)*
	*People with light skin*	*16 (1.1)*
	*Youth*	*11 (0.8)*
	*Women*	*11 (0.8)*
	*Women in menopause*	*9 (0.6)*
	*Smokers*	*4 (0.2)*
	*Men*	*3 (0.2)*
Heat actions	Hydrate	524 (37.6)
	Medical care	420 (30.2)
	Place person in cool location	399 (28.7)
	Cool the body	361 (25.9)
	Halt physical activity	120 (8.6)
	Adjust clothing	26 (1.9)
	*Provide local remedy*^d^	*70 (5.0)*
	*Provide medication*	*47 (3.4)*
	*Give food*	*12 (0.9)*
	*Do not go out*	*8 (0.6)*
	*Create green spaces*	*2 (0.1)*
	*Take a hot shower*	*1 (0.1)*
	*Lose weight*	*1 (0.1)*
Protective measures	Stay inside / visit cool areas	716 (51.4)
	Increase fluid consumption	688 (49.4)
	Adjust clothing	408 (29.3)
	Use fan / airconditioning	345 (24.8)
	Cool the body	182 (13.1)
	Avoid physical activity	121 (8.7)
	Use sunscreen	89 (6.4)
	Keep windows closed	24 (1.7)
	Adjust medication	23 (1.7)
	Adjust diet	19 (1.4)
	Limit alcohol consumption	2 (0.1)
	*Use local remedy*	*14 (1.0)*
	*Drink hot tea*	*1 (0.1)*
	*Take a hot shower*	*1 (0.1)*

^a^ Correct answers are presented in black, incorrect answers in blue and italic

^b^ Since death is not formally a symptom but a health outcome, we did not include it in the list of correct answers

^c^ Skin cancer was categorized under ‘skin problems’. Cancer without further specification was considered an incorrect answer

^d^ The answer ‘provide local remedy’ was only given by respondents in Tunisia, and refers to a traditional recipe extracted from trees (orange flower water). Since we do not have evidence on its effectiveness, we consider it an incorrect answer

### Differences in heatwave-protective knowledge and behaviour between countries

Additional file 2 shows the result in heatwave-protective knowledge for each of the countries separately. There are some notable differences in the proportion of respondents that provided a certain answer, such as: dehydration-related problems as a symptom varied from 16.9% in Georgia to 59.4% in Israel; babies/children as a risk group was relatively low in Georgia compared to the other two countries (23.9, 58.3, 59.0% in Georgia, Tunisia and Israel, respectively); providing a local remedy as a heat action was only mentioned by respondents in Tunisia (16.8%), and consisted of consuming orange flower water; the proportion of respondents who reported staying inside / visiting cool areas varied from 38.1% in Israel to 61.6% in Tunisia.

We asked respondents in Tunisia and Georgia whether, during the last heatwave, they had applied one or more of the protective measures they were able to name (Table 3). In Israel, due to the web format of the survey, the respondents were instead asked the extent to which they had applied protective measures from a predetermined list. For comparability, we only included answers from respondents who were able to name the respective measure in the open question on protective measures, and we considered protective measures for which they answered ‘always’, ‘usually’ and ‘occasionally’ as ‘yes’. In Tunisia and Israel, each protective measure was applied during the last heatwave by over 90% of respondents who named it, with one exception, namely ‘adjust medication’ in Israel, with 66.7%. However, the latter was based on the results of only three respondents. Usage of protective measures was slightly lower in Georgia, where three measures were used by over 80% but less than 90% of respondents who named them: adjust clothing for 81.3%, cool the body for 82.0%, and use of sunscreen for 85.7% of respondents.

**Table 3:** Use of protective measures in study countries

Protective measure^**a**^	Tunisia	Georgia	Israel^**b**^
	%	%	%
Stay inside / visit cool areas	96.5	93.1	99.0
Increase fluid consumption	99.2	97.8	98.8
Adjust clothing	96.4	81.3	93.9
Use fan / airconditioning	93.8	96.4	97.8
Cool the body	100.0	82.0	95.2
Avoid physical activity	100.0	91.3	97.8
Use sunscreen	95.8	85.7	–
Keep windows closed	100.0	95.0	–
Adjust medication	100.0	90.0	66.7
Adjust diet	100.0	100.0	100.0

^a^ Only answers from respondents who were able to name the measure are included in the table

^b^ In Israel, ‘use sunscreen’ was not one of the measures in the predetermined list, and ‘keep windows closed’ was not given as an answer by any respondent

**Fig. 1:**
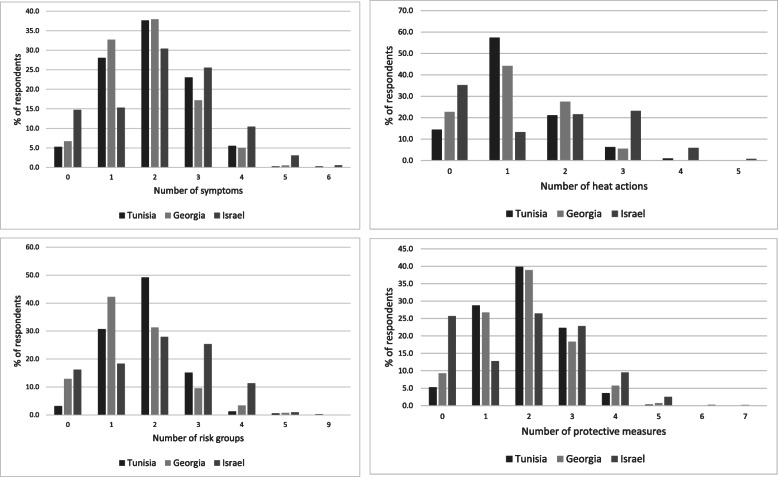
**a**. Number of correct symptoms mentioned by respondents. **b**. Number of correct risk groups mentioned by respondents. **c**. Number of correct heat actions mentioned by respondents. **d**. Number of correct protective measures mentioned by respondents

After aggregating the correct answers per question for each individual and comparing the averages between countries, most variation in the number of correct answers was seen in Israel. Here, we found the higher proportions of respondents who did not know any correct answer, as well as higher proportions of those who gave three or more correct answers, compared to the other two countries (Fig. 1). The mode (most prevalent number of correct answers) for all three countries in the questions on symptoms and protective measures was 2 (Fig. 1a, d). This was also the case for the question on risk groups, except for Georgia, where the mode was 1 (Fig. 1b). For the question on heat actions, the mode was 1 for Tunisia and Georgia, and 0 for Israel (Fig. 1c).

For each knowledge item, we calculated the average number of correct answers per country (Table 4). For symptoms, risk groups and heat actions, Israel is the country that scores on average highest and Georgia lowest. The variation was highest in Israel, as was also seen in Fig. 1.

**Table 4:** Mean number of knowledge level items in study countries

Knowledge item	Tunisia *Mean (sd)*	Georgia *Mean (sd)*	Israel *Mean (sd)*
Symptoms	2.0 (1.0)	1.8 (1.0)	2.1 (1.3)
Risk groups	1.8 (0.9)	1.5 (1.0)	2.0 (1.3)
Heat action	1.2 (0.8)	1.2 (0.8)	1.5 (1.4)
Protective measures	1.9 (0.9)	1.9 (1.1)	1.9 (1.2)

Also for the corrected analyses, Israel scores highest for all questions, except for protective measures, where Tunisia scored significantly higher (Table 5). For Symptoms and risk groups, Tunisia did not differ significantly from Israel, but for heat actions they were significantly lower. Georgia was always significantly lower than Israel, except for protective measures, where the difference was not significant.

**Table 5:** Differences in knowledge level between study countries, corrected for confounders

		Symptoms^**a**^		Risk groups^**a**^		Heat actions^**a**^		Protective measure^**a**^	
		b-value (CI)	*p*-value	b-value (CI)	*p*-value	b-value (CI)	*p*-value	b-value (CI)	*p*-value
Country^b^			.001		< .001		< .001		.066
	Tunisia	0.0 (−0.2 to 0.1)	.597	−0.1 (−0.3 to 0.1)	.333	−0.3 (−0.4 to −0.1)	.002	0.2 (0.0 to 0.4)	.032
	Georgia	−0.3 (− 0.4 to − 0.1)	< .001	−0.5 (− 0.7 to − 0.4)	< .001	−0.4 (− 0.6 to − 0.3)	< .001	0.0 (− 0.1 to 0.2)	.852
	Israel	Ref	Ref	Ref	Ref	Ref	Ref	Ref	Ref

^a^ Variables that were included in the analyses as confounders were gender, age, educational level, having children under 12 years old, employment status, having fasted in the previous year, taking medication for a chronic disease

^b^ Israel was used as the reference country for the analyses. The b-values of Tunisia and Georgia indicate how much higher or lower the correct number of answers on average was in that country, after correcting for confounders

### Information channels on heatwave-protective measures

In all three countries, the channel that respondents had most often used to look for information on heatwave-protective measures was television, although the proportions varied between the countries (17.5% in Tunisia, 32.1% in Georgia and 39% in Israel). In contrast, a large proportion of respondents in each country indicated not to have consulted any channel on heatwave-protective measures (53.2% in Tunisia, 24.2% in Georgia, 37.6% in Israel).

## Discussion

Our study provides important insights in heatwave-protective knowledge and behaviour in Tunisia, Georgia and Israel. The results show that in each of the countries, most of the respondents were able to name one or more symptoms associated to heatwave exposure, population risk groups for heatwaves, actions that should be taken when someone is overheated and heatwave-protective measures. Among the respondents who named certain protective measures, a large majority had also used them during the last heatwave, especially in Tunisia and Israel. Heatwave-protective knowledge was also significantly higher in those countries, compared to Georgia. These insights are valuable, as they allow to identify priority areas for risk communication and awareness raising activities in each of the countries.

The risk groups that were most often mentioned by respondents were elderly, babies/children and the physically ill. This is in line with previous population surveys on heatwave preparedness undertaken in Belgium, the Netherlands, Spain and Portugal [12, 13]. These studies were undertaken by the first author of this paper, using a similar questionnaire as the one used in this study, and a similar sampling strategy as the survey in Tunisia and Georgia. The European studies focused also on urban populations, but only on the capital of each country. The proportion of respondents in the European countries that was able to name elderly as a risk group varied between countries from 70 to 90%, versus on average 60% in the Neighbourhood countries. This could be due to the fact that the elderly comprises a much larger fraction of the population in European countries compared to the European Neighbourhood. This is plausible, since the results on the other groups are much more in line with each other: babies/children was reported by around 40–65% of respondents in the previous European studies, versus 48% in the European Neighbourhood, while the proportion that named physically ill was around 35% everywhere, except for Spain (16%) [12, 13]. Other vulnerable groups (e.g. people who work outdoors, people who use medication for a chronic disease, people who are socially isolated) are much less known, indicating a need for increasing awareness on which population groups are vulnerable to heat.

On average 50% of respondents in our study were able to name staying inside or visiting cool areas as a heatwave-protective measure, and an equally large group hydrating, although there was large variation between the three countries. The former average is similar to the results from previous studies in Belgium, the Netherlands, Spain and Portugal using a similar methodology, while the latter is lower (proportions in Europe varied from 60 to 80%) [12, 13]. The most commonly named heat-related symptom was dehydration by 39% of respondents from the three countries in our study, and the most common action that people described to counter overheating was hydrating by 38%. The proportions for these relatively straightforward answers are somewhat low. Furthermore, a significant proportion of respondents provided incorrect answers, such as nosebleed as a symptom of overheating, which did not occur in the previous study in Spain and Portugal, and providing medication as an action to take when someone is overheated. Combined, these results imply that there is a need to increase knowledge on heatwave protection, in particular for symptoms and actions to counter overheating. This could be achieved by incorporating National Heat Health Action Plans according to the framework by the World Health Organization [34], as respondents from countries that have such plans already in place seem to have higher heatwave-protective knowledge.

When comparing the climates of the three countries in our study, we see that Tunisia and Israel are very similar (average high temperatures reaching values of 32.4 and 32.7 °C during the warmest summer month, respectively) and much warmer than Georgia (average high temperature of 27.0 °C in August) [35]. This is a likely explanation for the finding that heatwave-protective knowledge was significantly lower in Georgia than in the other two countries, since people in Tunisia and Israel may be more accustomed to heatwaves and likely have more experience in recognising symptoms and risk groups for heatwaves, and in countering overheating. This is also implied by the fact that respondents in Tunisia and Israel consulted channels with information on heatwave-protective knowledge less frequently than respondents in Georgia. Nonetheless, as temperatures are set to increase in the coming years and the frequency, duration and intensity of heatwaves will be affected [2], improving heatwave-protective knowledge in Georgia is equally important as it is in Tunisia and Israel. Between Tunisia and Israel, knowledge on protective measures was higher in Tunisia while knowledge on countering overheating was higher in Israel. This finding implies that the Tunisian population tends to focus more on prevention, and the Israeli population on response, although these conclusions would need to be substantiated with further evidence.

Individual disaster preparedness requires appropriate knowledge on how to prepare, protect and adapt oneself. However, it also requires a consequential change in behaviour, which is a major field of research [36, 37]. A study on sea level rise and flooding suggested that risk communication should integrate information on how to adapt behaviour [38]. A study on heat-related knowledge, attitude and practices among pilgrims in the 2017 Hajj Mass gathering found that, despite many respondents having a good knowledge, there was a reluctance to apply protective measures or to hydrate properly [39]. In contrast, our study found that among respondents with knowledge on heatwave-protective measures, the overall majority indicated that they had also applied these measures during the last heatwave. The higher applications into behaviour in Tunisia and Israel versus Georgia was likely caused by the greater severity of heatwaves in the former two countries, which increases the necessity of integrating protective behaviour in daily life.

Previous studies showed that people tend to underestimate their own risk, even if they belong to a risk group [12, 40], and that heatwave-protective knowledge is significantly associated with personal characteristics such as gender, age and educational level [12, 41]. It is of interest to identify risk factors for heatwave-protective knowledge in our study countries, which we will address in upcoming, country-specific publications.

Our study had several strengths. We included a sample size large enough to represent each of the study countries. Furthermore, by including three cities per country, we ensured that the results do not only reflect the population of the biggest city, but of a wider range of urban residents in different climate zones. By sampling different neighbourhoods within each city, we also ensured variation in socio-economic background of respondents. All enumerators that participated in the street survey were trained by the lead researchers and used the same protocol and questionnaire, to ensure comparability between different enumerators as much as possible. The survey in Israel was translated to Hebrew as well as Arabic, to include respondents from both main language groups.

This study had some limitations. First, there was a difference in data collection methodology between Tunisia and Georgia on the one hand, and Israel on the other (street survey and internet survey, respectively), which could possibly have influenced answers of respondents. Second, the street survey only included respondents who the survey team encountered on the street at a given time, and were less likely to include persons with limited mobility, who might be especially vulnerable to the negative effects of heatwaves. Third, due to language issues, the data collection team consisted of different enumerators in Tunisia and Georgia. However, all enumerators were trained by the lead researchers and used the same protocol and questionnaire, to ensure comparability. Finally, the internet survey excluded individuals without access to internet, but since internet penetration is 84% in Israel [42], we expect the impact of this to be limited.

## Conclusion

Our results indicate that there is a need to further improve heatwave-protective knowledge in Tunisia, Georgia and Israel. Since none of the countries currently possess a National Heat Health Action Plan, and such plans usually contain dedicated awareness raising activities, this seems like a suitable strategy to increase this vital knowledge. We also found that, for those individuals with knowledge on heat-protective measures, the overall majority indicates that they apply this knowledge into behaviour during heatwaves. This further emphasises the importance of improving knowledge on heatwave-protective measures among the populations of these countries.

## Supplementary Information

**Additional file 1:** Study questionnaire.

**Additional file 2:** Heatwave-protective answers given by respondents per country.

## Data Availability

The data supporting the results of this article are available upon request from the corresponding author.
