# Supplementary material for: Heatwave-protective knowledge and behaviour among urban populations: a multi-country study in Tunisia, Georgia and Israel
Source: BMC Public Health. 2021 May 1;21:834. doi: 10.1186/s12889-021-10865-y (PMC8088049; doi:10.1186/s12889-021-10865-y)
Supplement: Supplementary file 1 — Additional file 1: Study questionnaire. [file 12889_2021_10865_MOESM1_ESM.docx]

**Heatwave-protective knowledge and behaviour among urban populations: a multi-country study in Tunisia, Georgia and Israel**

Joris Adriaan Frank van Loenhout^1^, Kirsten Vanderplanken^1^, Tamari Kashibadze^2^, Nia Giuashvili^2^, Amiran Gamkrelidze^2^, Maya Siman-Tov^3^, Bruria Adini^3^, Debarati Guha-Sapir^1^

1) Centre for Research on the Epidemiology of Disasters, Institute of Health and Society, Université catholique de Louvain, Brussels, Belgium

2) L. Sakvarelidze National Center for Disease Control and Public Health (NCDC), Ministry of IDP from the occupied territories, Labour, Health and Social Affairs of Georgia, Tbilisi, Georgia

3) Emergency Management and Disaster Medicine department, School of Public Health, Sackler Faculty of Medicine, Tel Aviv University, Tel Aviv, Israel

**Corresponding Author**

Joris Adriaan Frank van Loenhout

Centre for Research on the Epidemiology of Disasters

Institute of Health and Society

Université catholique de Louvain

Clos Chapelle-aux-Champs 30

1200 Brussels, Belgium

[Joris.vanloenhout@uclouvain.be](mailto:Joris.vanloenhout@uclouvain.be)

0031-646189350

**Additional file I: Study questionnaire**

1. **DEMOGRAPHY**
2. Do you currently live in CITY?

No 🡪 **If no, not eligible for this study!**  Yes

**🡪 If yes:** Have you lived here for over 5 years?

No  Yes

1. What is your age? **If under 18** 🡪 **not eligible for this study**
2. Gender

Male  Female  Other

1. What is your educational level?

None

Completed primary education

Completed secondary education

Completed vocational or professional education

Completed college or university studies

1. Do you have children aged 12 years or under?

No  Yes

1. What is your current employment status?

Student

Employed or self-employed

Unemployed

Retired

Housewife

Other

6a. **🡪 If “employed or self-employed”:** Does your job require you to work mainly outdoors between 10 AM and 3 PM?

No  Yes

6b. **🡪 If “employed or self-employed”:** Does your job include taking care of other people? (e.g. nursery, school or elderly care centre)

No  Yes

1. Have you participated in a fast that lasted multiple, consecutive days between May and September (e.g. religious fast or diet)?

No  Yes

1. Do you take a medication every day for a chronic disease? (e.g. high blood pressure, diabetes, asthma)

No  Yes

1. **AWARENESS ABOUT HEALTH IMPACT OF HEATWAVES**
2. Can you name some of the symptoms people may experience due to a heatwave? **(open question and multiple answers possible, do NOT present answers to respondent)**

1. Can you name some groups of people who are at a higher risk to suffer from health effects due to a heatwave? **(open question and multiple answers possible, do NOT present answers to respondent)**

1. **KNOWLEDGE ABOUT AND BEHAVIOUR FOR REDUCING HEALTH IMPACT OF HEATWAVES**
2. Do you know what to do when someone suffers from a heat stroke/overheating? **(open question and multiple answers possible, do NOT present answers to respondent)**

1. a) Are you familiar with some measures you can take to protect yourself from a heatwave? **(open question and multiple answers possible, do NOT present answers to respondent)**


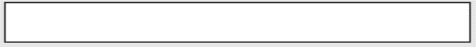


***ONLY ASKED IN TUNISIA AND GEORGIA***

b) 🡪 **After listing all measures, ask for each of the measures that were given as answers:** did you implement this measure this during the last summer?

Used during last summer?

Yes  No

1. Which of the following information sources did you consult during the past summer to look for information on measures to protect yourself from a heatwave? **(open question and multiple answers possible, do NOT present answers to respondent)**

***ONLY ASKED IN ISRAEL***

1. During the last heatwave, indicate the extent to which you:

|  | Always | Usually | Occasionally | Never | Not relevant |
| --- | --- | --- | --- | --- | --- |
| Drank more water |  |  |  |  |  |
| Stayed inside during hottest hours |  |  |  |  |  |
| Stayed in a park or green area |  |  |  |  |  |
| Wore clothing with light colours and materials |  |  |  |  |  |
| Cooled your body by taking a shower or go swimming |  |  |  |  |  |
| Adjusted your medication |  |  |  |  |  |
| Used a fan or air conditioning |  |  |  |  |  |
| Took medication to lower your body temperature |  |  |  |  |  |
| Wore a hat |  |  |  |  |  |
| Limited physical activity |  |  |  |  |  |
| Closed windows and curtains |  |  |  |  |  |
| Ate light meals |  |  |  |  |  |

1. Which of the following information sources did you use during the past summer to look for information on measures to protect yourself from a heatwave? (multiple anwers possible)

Government social media or website

Government brochure or poster

Television

Radio

Newspaper or online news website

Doctor or other health professional

Relatives/friends

I did not consult any source for information on protective measures

Other:

**Additional file II. Heatwave-protective answers given by respondents per country**

| **Most prevalent heatwave-protective answers given by respondents in Tunisia** | | | | | |
| --- | --- | --- | --- | --- | --- |
| **Question** | **Answer** | **N (%)** | **Question** | **Answer** | **N (%)** |
| Symptoms |  |  | Risk groups |  |  |
|  | Dehydration-related problems | 136 (32.6) |  | Elderly | 299 (71.7) |
|  | Headache | 145 (34.8) |  | Babies / children | 243 (58.3) |
|  | Exhaustion | 175 (42.0) |  | Physically ill | 66 (15.8) |
|  | Thermoregulation-related problems | 91 (21.8) |  | Pregnant women | 12 (2.9) |
|  | Dizziness / fainting | 30 (7.2) |  | People who perform physical effort / work mainly outdoors | 75 (18.0) |
|  | Cardiovascular problems | 56 (13.4) |  | People who use medication for chronic disease | 49 (11.8) |
|  | Skin problems | 94 (22.5) |  | Obese | 0 (0.0) |
|  | Respiratory problems | 41 (9.8) |  | Handicapped or limited mobility | 4 (1.0) |
|  | Behavioural and cognitive problems | 25 (6.0) |  | Socially isolated | 10 (2.4) |
|  | Gastrointestinal problems | 9 (2.2) |  | Mentally ill | 3 (0.7) |
|  | Neuromuscular problems | 11 (2.6) |  | People with lower socio-economic status | 5 (1.2) |
|  | Other general problems | 3 (0.7) |  | Substance abusers | 0 (0.0) |
|  | Eye problems | 6 (1.4) |  | *General public* | *1 (0.2)* |
|  | *Death* | *6 (1.4)* |  | *People with light skin* | *9 (2.2)* |
|  | *Nosebleed* | *10 (2.4)* |  | *Youth* | *5 (1.2)* |
|  | *Allergy* | *4 (1.0)* |  | *Women* | *0 (0.0)* |
|  | *Cancer* | *6 (1.4)* |  | *Women in menopause* | *0 (0.0)* |
|  | *Diabetes* | *6 (1.4)* |  | *Smokers* | *0 (0.0)* |
|  | *Seizures* | *0 (0.0)* |  | *Men* | *0 (0.0)* |
| Heat actions | |  | Protective measures | |  |
|  | Hydrate | 82 (19.7) |  | Stay inside / visit cool areas | 257 (61.6) |
|  | Medical care | 239 (57.3) |  | Increase fluid consumption | 128 (30.7) |
|  | Place person in cool location | 51 (12.2) |  | Adjust clothing | 168 (40.3) |
|  | Cool the body | 126 (30.2) |  | Use fan / airconditioning | 96 (23.0) |
|  | Halt physical activity | 4 (1.0) |  | Cool the body | 79 (18.9) |
|  | Adjust clothing | 7 (1.7) |  | Avoid physical activity | 5 (1.2) |
|  | *Provide local remedy* | *70 (16.8)* |  | Use sunscreen | 48 (11.5) |
|  | *Provide medication* | *32 (7.7)* |  | Keep windows closed | 4 (1.0) |
|  | *Give food* | *1 (0.2)* |  | Adjust medication | 8 (1.9) |
|  | *Do not go out* | *0 (0.0)* |  | Adjust diet | 3 (0.7) |
|  | *Create green spaces* | *0 (0.0)* |  | Limit alcohol consumption | 0 (0.0) |
|  | *Take a hot shower* | *0 (0.0)* |  | *Use local remedy* | *14 (3.4)* |
|  | *Loose weight* | *0 (0.0)* |  | *Drink hot tea* | *0 (0.0)* |
|  |  |  |  | *Take a hot shower* | *0 (0.0)* |

| **Most prevalent heatwave-protective answers given by respondents in Georgia** | | | | | |
| --- | --- | --- | --- | --- | --- |
| **Question** | **Answer** | **N (%)** | **Question** | **Answer** | **N (%)** |
| Symptoms |  |  | Risk groups |  |  |
|  | Dehydration-related problems | 71 (16.9) |  | Elderly | 243 (58.0) |
|  | Headache | 171 (40.8) |  | Babies / children | 100 (23.9) |
|  | Exhaustion | 120 (28.6) |  | Physically ill | 141 (33.7) |
|  | Thermoregulation-related problems | 85 (20.3) |  | Pregnant women | 32 (7.6) |
|  | Dizziness / fainting | 41 (9.8) |  | People who perform physical effort / work mainly outdoors | 48 (11.5) |
|  | Cardiovascular problems | 105 (25.1) |  | People who use medication for chronic disease | 21 (5.0) |
|  | Skin problems | 60 (14.3) |  | Obese | 8 (1.9) |
|  | Respiratory problems | 62 (14.8) |  | Handicapped or limited mobility | 14 (3.3) |
|  | Behavioural and cognitive problems | 15 (3.6) |  | Socially isolated | 13 (3.1) |
|  | Gastrointestinal problems | 20 (4.8) |  | Mentally ill | 10 (2.4) |
|  | Neuromuscular problems | 10 (2.4) |  | People with lower socio-economic status | 0 (0.0) |
|  | Other general problems | 4 (1.0) |  | Substance abusers | 0 (0.0) |
|  | Eye problems | 1 (0.2) |  | *General public* | *10 (2.4)* |
|  | *Death* | *0 (0.0)* |  | *People with light skin* | *0 (0.0)* |
|  | *Nosebleed* | *9 (2.1)* |  | *Youth* | *5 (1.2)* |
|  | *Allergy* | *0 (0.0)* |  | *Women* | *5 (1.2)* |
|  | *Cancer* | *0 (0.0)* |  | *Women in menopause* | *0 (0.0)* |
|  | *Diabetes* | *0 (0.0)* |  | *Smokers* | *0 (0.0)* |
|  | *Seizures* | *0 (0.0)* |  | *Men* | *2 (0.5)* |
| Heat actions | |  | Protective measures | |  |
|  | Hydrate | 153 (36.5) |  | Stay inside / visit cool areas | 247 (58.9) |
|  | Medical care | 72 (17.2) |  | Increase fluid consumption | 224 (53.5) |
|  | Place person in cool location | 178 (42.5) |  | Adjust clothing | 75 (17.9) |
|  | Cool the body | 41 (9.8) |  | Use fan / airconditioning | 110 (26.3) |
|  | Halt physical activity | 44 (10.5) |  | Cool the body | 61 (14.6) |
|  | Adjust clothing | 0 (0.0) |  | Avoid physical activity | 23 (5.5) |
|  | *Provide local remedy* | *0 (0.0)* |  | Use sunscreen | 7 (1.7) |
|  | *Provide medication* | *10 (2.4)* |  | Keep windows closed | 20 (4.8) |
|  | *Give food* | *2 (0.5)* |  | Adjust medication | 11 (2.6) |
|  | *Do not go out* | *8 (1.9)* |  | Adjust diet | 8 (1.9) |
|  | *Create green spaces* | *2 (0.5)* |  | Limit alcohol consumption | 1 (0.2) |
|  | *Take a hot shower* | *1 (0.2)* |  | *Use local remedy* | *0 (0.0)* |
|  | *Loose weight* | *1 (0.2)* |  | *Drink hot tea* | *1 (0.2)* |
|  |  |  |  | *Take a hot shower* | *0 (0.0)* |

| **Most prevalent heatwave-protective answers given by respondents in Israel** | | | | | |
| --- | --- | --- | --- | --- | --- |
| **Question** | **Answer** | **N (%)** | **Question** | **Answer** | **N (%)** |
| Symptoms |  |  | Risk groups |  |  |
|  | Dehydration-related problems | 330 (59.4) |  | Elderly | 289 (52.0) |
|  | Headache | 115 (20.7) |  | Babies / children | 328 (59.0) |
|  | Exhaustion | 115 (20.7) |  | Physically ill | 283 (50.9) |
|  | Thermoregulation-related problems | 165 (29.7) |  | Pregnant women | 126 (22.7) |
|  | Dizziness / fainting | 159 (28.6) |  | People who perform physical effort / work mainly outdoors | 29 (5.2) |
|  | Cardiovascular problems | 42 (7.6) |  | People who use medication for chronic disease | 1 (0.2) |
|  | Skin problems | 48 (8.6) |  | Obese | 31 (5.6) |
|  | Respiratory problems | 66 (11.9) |  | Handicapped or limited mobility | 15 (2.7) |
|  | Behavioural and cognitive problems | 107 (19.2) |  | Socially isolated | 4 (0.7) |
|  | Gastrointestinal problems | 21 (3.8) |  | Mentally ill | 0 (0.0) |
|  | Neuromuscular problems | 0 (0.0) |  | People with lower socio-economic status | 5 (0.9) |
|  | Other general problems | 10 (1.8) |  | Substance abusers | 1 (0.2) |
|  | Eye problems | 6 (1.1) |  | *General public* | *101 (18.2)* |
|  | *Death* | *30 (5.4)* |  | *People with light skin* | *7 (1.3)* |
|  | *Nosebleed* | *1 (0.2)* |  | *Youth* | *1 (0.2)* |
|  | *Allergy* | *4 (0.7)* |  | *Women* | *6 (1.1)* |
|  | *Cancer* | *2 (0.4)* |  | *Women in menopause* | *9 (1.6)* |
|  | *Diabetes* | *0 (0.0)* |  | *Smokers* | *4 (0.7)* |
|  | *Seizures* | *2 (0.4)* |  | *Men* | *1 (0.2)* |
| Heat actions | |  | Protective measures | |  |
|  | Hydrate | 289 (52.0) |  | Stay inside / visit cool areas | 212 (38.1) |
|  | Medical care | 109 (19.6) |  | Increase fluid consumption | 336 (60.4) |
|  | Place person in cool location | 170 (30.6) |  | Adjust clothing | 165 (29.7) |
|  | Cool the body | 194 (34.9) |  | Use fan / airconditioning | 139 (25.0) |
|  | Halt physical activity | 72 (12.9) |  | Cool the body | 42 (7.6) |
|  | Adjust clothing | 19 (3.4) |  | Avoid physical activity | 93 (16.7) |
|  | *Provide local remedy* | *0 (0.0)* |  | Use sunscreen | 34 (6.1) |
|  | *Provide medication* | *5 (0.9)* |  | Keep windows closed | 0 (0.0) |
|  | *Give food* | *9 (1.6)* |  | Adjust medication | 4 (0.7) |
|  | *Do not go out* | *0 (0.0)* |  | Adjust diet | 8 (1.4) |
|  | *Create green spaces* | *0 (0.0)* |  | Limit alcohol consumption | 1 (0.2) |
|  | *Take a hot shower* | *0 (0.0)* |  | *Use local remedy* | *0 (0.0)* |
|  | *Loose weight* | *0 (0.0)* |  | *Drink hot tea* | *0 (0.0)* |
|  |  |  |  | *Take a hot shower* | *0 (0.0)* |
