# Supplementary material for: Heatwave-protective knowledge and behaviour among urban populations: a multi-country study in Tunisia, Georgia and Israel
Source: BMC Public Health. 2021 May 1;21:834. doi: 10.1186/s12889-021-10865-y (PMC8088049; doi:10.1186/s12889-021-10865-y)
Supplement: Supplementary file 2 — Additional file 2: Heatwave-protective answers given by respondents per country. [file 12889_2021_10865_MOESM2_ESM.docx]

**Heatwave-protective knowledge and behaviour among urban populations: a multi-country study in Tunisia, Georgia and Israel**

Joris Adriaan Frank van Loenhout^1^, Kirsten Vanderplanken^1^, Tamari Kashibadze^2^, Nia Giuashvili^2^, Amiran Gamkrelidze^2^, Maya Siman-Tov^3^, Bruria Adini^3^, Debarati Guha-Sapir^1^

1) Centre for Research on the Epidemiology of Disasters, Institute of Health and Society, Université catholique de Louvain, Brussels, Belgium

2) L. Sakvarelidze National Center for Disease Control and Public Health (NCDC), Ministry of IDP from the occupied territories, Labour, Health and Social Affairs of Georgia, Tbilisi, Georgia

3) Emergency Management and Disaster Medicine department, School of Public Health, Sackler Faculty of Medicine, Tel Aviv University, Tel Aviv, Israel

**Corresponding Author**

Joris Adriaan Frank van Loenhout

Centre for Research on the Epidemiology of Disasters

Institute of Health and Society

Université catholique de Louvain

Clos Chapelle-aux-Champs 30

1200 Brussels, Belgium

[Joris.vanloenhout@uclouvain.be](mailto:Joris.vanloenhout@uclouvain.be)

0031-646189350

**Additional file II. Heatwave-protective answers given by respondents per country**

| **Most prevalent heatwave-protective answers given by respondents in Tunisia** | | | | | |
| --- | --- | --- | --- | --- | --- |
| **Question** | **Answer** | **N (%)** | **Question** | **Answer** | **N (%)** |
| Symptoms |  |  | Risk groups |  |  |
|  | Dehydration-related problems | 136 (32.6) |  | Elderly | 299 (71.7) |
|  | Headache | 145 (34.8) |  | Babies / children | 243 (58.3) |
|  | Exhaustion | 175 (42.0) |  | Physically ill | 66 (15.8) |
|  | Thermoregulation-related problems | 91 (21.8) |  | Pregnant women | 12 (2.9) |
|  | Dizziness / fainting | 30 (7.2) |  | People who perform physical effort / work mainly outdoors | 75 (18.0) |
|  | Cardiovascular problems | 56 (13.4) |  | People who use medication for chronic disease | 49 (11.8) |
|  | Skin problems | 94 (22.5) |  | Obese | 0 (0.0) |
|  | Respiratory problems | 41 (9.8) |  | Handicapped or limited mobility | 4 (1.0) |
|  | Behavioural and cognitive problems | 25 (6.0) |  | Socially isolated | 10 (2.4) |
|  | Gastrointestinal problems | 9 (2.2) |  | Mentally ill | 3 (0.7) |
|  | Neuromuscular problems | 11 (2.6) |  | People with lower socio-economic status | 5 (1.2) |
|  | Other general problems | 3 (0.7) |  | Substance abusers | 0 (0.0) |
|  | Eye problems | 6 (1.4) |  | *General public* | *1 (0.2)* |
|  | *Death* | *6 (1.4)* |  | *People with light skin* | *9 (2.2)* |
|  | *Nosebleed* | *10 (2.4)* |  | *Youth* | *5 (1.2)* |
|  | *Allergy* | *4 (1.0)* |  | *Women* | *0 (0.0)* |
|  | *Cancer* | *6 (1.4)* |  | *Women in menopause* | *0 (0.0)* |
|  | *Diabetes* | *6 (1.4)* |  | *Smokers* | *0 (0.0)* |
|  | *Seizures* | *0 (0.0)* |  | *Men* | *0 (0.0)* |
| Heat actions | |  | Protective measures | |  |
|  | Hydrate | 82 (19.7) |  | Stay inside / visit cool areas | 257 (61.6) |
|  | Medical care | 239 (57.3) |  | Increase fluid consumption | 128 (30.7) |
|  | Place person in cool location | 51 (12.2) |  | Adjust clothing | 168 (40.3) |
|  | Cool the body | 126 (30.2) |  | Use fan / airconditioning | 96 (23.0) |
|  | Halt physical activity | 4 (1.0) |  | Cool the body | 79 (18.9) |
|  | Adjust clothing | 7 (1.7) |  | Avoid physical activity | 5 (1.2) |
|  | *Provide local remedy* | *70 (16.8)* |  | Use sunscreen | 48 (11.5) |
|  | *Provide medication* | *32 (7.7)* |  | Keep windows closed | 4 (1.0) |
|  | *Give food* | *1 (0.2)* |  | Adjust medication | 8 (1.9) |
|  | *Do not go out* | *0 (0.0)* |  | Adjust diet | 3 (0.7) |
|  | *Create green spaces* | *0 (0.0)* |  | Limit alcohol consumption | 0 (0.0) |
|  | *Take a hot shower* | *0 (0.0)* |  | *Use local remedy* | *14 (3.4)* |
|  | *Loose weight* | *0 (0.0)* |  | *Drink hot tea* | *0 (0.0)* |
|  |  |  |  | *Take a hot shower* | *0 (0.0)* |

| **Most prevalent heatwave-protective answers given by respondents in Georgia** | | | | | |
| --- | --- | --- | --- | --- | --- |
| **Question** | **Answer** | **N (%)** | **Question** | **Answer** | **N (%)** |
| Symptoms |  |  | Risk groups |  |  |
|  | Dehydration-related problems | 71 (16.9) |  | Elderly | 243 (58.0) |
|  | Headache | 171 (40.8) |  | Babies / children | 100 (23.9) |
|  | Exhaustion | 120 (28.6) |  | Physically ill | 141 (33.7) |
|  | Thermoregulation-related problems | 85 (20.3) |  | Pregnant women | 32 (7.6) |
|  | Dizziness / fainting | 41 (9.8) |  | People who perform physical effort / work mainly outdoors | 48 (11.5) |
|  | Cardiovascular problems | 105 (25.1) |  | People who use medication for chronic disease | 21 (5.0) |
|  | Skin problems | 60 (14.3) |  | Obese | 8 (1.9) |
|  | Respiratory problems | 62 (14.8) |  | Handicapped or limited mobility | 14 (3.3) |
|  | Behavioural and cognitive problems | 15 (3.6) |  | Socially isolated | 13 (3.1) |
|  | Gastrointestinal problems | 20 (4.8) |  | Mentally ill | 10 (2.4) |
|  | Neuromuscular problems | 10 (2.4) |  | People with lower socio-economic status | 0 (0.0) |
|  | Other general problems | 4 (1.0) |  | Substance abusers | 0 (0.0) |
|  | Eye problems | 1 (0.2) |  | *General public* | *10 (2.4)* |
|  | *Death* | *0 (0.0)* |  | *People with light skin* | *0 (0.0)* |
|  | *Nosebleed* | *9 (2.1)* |  | *Youth* | *5 (1.2)* |
|  | *Allergy* | *0 (0.0)* |  | *Women* | *5 (1.2)* |
|  | *Cancer* | *0 (0.0)* |  | *Women in menopause* | *0 (0.0)* |
|  | *Diabetes* | *0 (0.0)* |  | *Smokers* | *0 (0.0)* |
|  | *Seizures* | *0 (0.0)* |  | *Men* | *2 (0.5)* |
| Heat actions | |  | Protective measures | |  |
|  | Hydrate | 153 (36.5) |  | Stay inside / visit cool areas | 247 (58.9) |
|  | Medical care | 72 (17.2) |  | Increase fluid consumption | 224 (53.5) |
|  | Place person in cool location | 178 (42.5) |  | Adjust clothing | 75 (17.9) |
|  | Cool the body | 41 (9.8) |  | Use fan / airconditioning | 110 (26.3) |
|  | Halt physical activity | 44 (10.5) |  | Cool the body | 61 (14.6) |
|  | Adjust clothing | 0 (0.0) |  | Avoid physical activity | 23 (5.5) |
|  | *Provide local remedy* | *0 (0.0)* |  | Use sunscreen | 7 (1.7) |
|  | *Provide medication* | *10 (2.4)* |  | Keep windows closed | 20 (4.8) |
|  | *Give food* | *2 (0.5)* |  | Adjust medication | 11 (2.6) |
|  | *Do not go out* | *8 (1.9)* |  | Adjust diet | 8 (1.9) |
|  | *Create green spaces* | *2 (0.5)* |  | Limit alcohol consumption | 1 (0.2) |
|  | *Take a hot shower* | *1 (0.2)* |  | *Use local remedy* | *0 (0.0)* |
|  | *Loose weight* | *1 (0.2)* |  | *Drink hot tea* | *1 (0.2)* |
|  |  |  |  | *Take a hot shower* | *0 (0.0)* |

| **Most prevalent heatwave-protective answers given by respondents in Israel** | | | | | |
| --- | --- | --- | --- | --- | --- |
| **Question** | **Answer** | **N (%)** | **Question** | **Answer** | **N (%)** |
| Symptoms |  |  | Risk groups |  |  |
|  | Dehydration-related problems | 330 (59.4) |  | Elderly | 289 (52.0) |
|  | Headache | 115 (20.7) |  | Babies / children | 328 (59.0) |
|  | Exhaustion | 115 (20.7) |  | Physically ill | 283 (50.9) |
|  | Thermoregulation-related problems | 165 (29.7) |  | Pregnant women | 126 (22.7) |
|  | Dizziness / fainting | 159 (28.6) |  | People who perform physical effort / work mainly outdoors | 29 (5.2) |
|  | Cardiovascular problems | 42 (7.6) |  | People who use medication for chronic disease | 1 (0.2) |
|  | Skin problems | 48 (8.6) |  | Obese | 31 (5.6) |
|  | Respiratory problems | 66 (11.9) |  | Handicapped or limited mobility | 15 (2.7) |
|  | Behavioural and cognitive problems | 107 (19.2) |  | Socially isolated | 4 (0.7) |
|  | Gastrointestinal problems | 21 (3.8) |  | Mentally ill | 0 (0.0) |
|  | Neuromuscular problems | 0 (0.0) |  | People with lower socio-economic status | 5 (0.9) |
|  | Other general problems | 10 (1.8) |  | Substance abusers | 1 (0.2) |
|  | Eye problems | 6 (1.1) |  | *General public* | *101 (18.2)* |
|  | *Death* | *30 (5.4)* |  | *People with light skin* | *7 (1.3)* |
|  | *Nosebleed* | *1 (0.2)* |  | *Youth* | *1 (0.2)* |
|  | *Allergy* | *4 (0.7)* |  | *Women* | *6 (1.1)* |
|  | *Cancer* | *2 (0.4)* |  | *Women in menopause* | *9 (1.6)* |
|  | *Diabetes* | *0 (0.0)* |  | *Smokers* | *4 (0.7)* |
|  | *Seizures* | *2 (0.4)* |  | *Men* | *1 (0.2)* |
| Heat actions | |  | Protective measures | |  |
|  | Hydrate | 289 (52.0) |  | Stay inside / visit cool areas | 212 (38.1) |
|  | Medical care | 109 (19.6) |  | Increase fluid consumption | 336 (60.4) |
|  | Place person in cool location | 170 (30.6) |  | Adjust clothing | 165 (29.7) |
|  | Cool the body | 194 (34.9) |  | Use fan / airconditioning | 139 (25.0) |
|  | Halt physical activity | 72 (12.9) |  | Cool the body | 42 (7.6) |
|  | Adjust clothing | 19 (3.4) |  | Avoid physical activity | 93 (16.7) |
|  | *Provide local remedy* | *0 (0.0)* |  | Use sunscreen | 34 (6.1) |
|  | *Provide medication* | *5 (0.9)* |  | Keep windows closed | 0 (0.0) |
|  | *Give food* | *9 (1.6)* |  | Adjust medication | 4 (0.7) |
|  | *Do not go out* | *0 (0.0)* |  | Adjust diet | 8 (1.4) |
|  | *Create green spaces* | *0 (0.0)* |  | Limit alcohol consumption | 1 (0.2) |
|  | *Take a hot shower* | *0 (0.0)* |  | *Use local remedy* | *0 (0.0)* |
|  | *Loose weight* | *0 (0.0)* |  | *Drink hot tea* | *0 (0.0)* |
|  |  |  |  | *Take a hot shower* | *0 (0.0)* |
